# Supplementary material for: Amaryllidaceae Alkaloids from Clivia miniata (Lindl.) Bosse (Amaryllidaceae): Isolation, Structural Elucidation, and Biological Activity
Source: Plants (Basel). 2022 Nov 10;11(22):3034. doi: 10.3390/plants11223034 (PMC9692855; doi:10.3390/plants11223034)
Supplement: Supplementary file 1 [file plants-11-03034-s001.zip › plants-2021762-supplementary.pdf]

SUPPLEMENTARY MATERIALS FOR:

Amaryllidaceae alkaloids from *Clivia miniata* (Lindl.) Bosse (Amaryllidaceae): isolation, structural elucidation, biological activity

Marcela Šafratová[1], Jana Křoustková[1], Negar Maafi[1], Daniela Suchánková[1], Rudolf Vrabec[1], Jakub Chlebek[1], Jiří Kuneš[2], Lubomír Opletal[1], Franz Bucar[3], and Lucie Cahlíková[1,*]

[1] Department of Pharmacognosy and Pharmaceutical Botany, Faculty of Pharmacy, Charles University, Heyrovskeho 1203, Hradec Kralove 500 05, Czech Republic

[2] Department of Bioorganic and Organic Chemistry, Faculty of Pharmacy, Charles University, Heyrovskeho 1203, Hradec Kralove 500 05, Czech Republic

[3] Institute of Pharmaceutical Sciences, University of Graz, Beethovenstraße 8, 8010 Graz, Austria

* Correspondence: cahlikova@faf.cuni.cz

Table of contents

Figure S1. HRESIMS spectrum of **3** S3

Figure S2. ^{1}H NMR spectrum of **3** analyzed in pyridine-d_5 with expanded parts with crucial *J*-couplings highlighted S4

Figure S3. ^{13}C NMR spectrum of **3** analyzed in pyridine-d_5........ S5

Figure S4. gHSQC spectrum of **3** analyzed in pyridine-d_5........ S5

Figure S5. gCOSY spectrum of **3** analyzed in pyridine-d_5........ S6

Figure S6. H2BC spectrum of **3** analyzed in pyridine-d_5........ S6

Figure S7. gHMBCAD spectrum of **3** analyzed in pyridine-d_5........ S7

Figure S8. NOESY spectrum of **3** analyzed in pyridine-d_5........ S7

Table S1. Comparison of ^{1}H NMR data of unit A of **3** with the structurally related cliniatine C........ S8

Table S2. Unit B ^{1}H and ^{13}C NMR data compared to C5-epimers from synthesis of narseronine........ S9

Biological assay in detail........ S11

hAChE and hBuChE inhibition assay........ S11

References........ S11

Figure S1. HRESIMS spectrum of **3**

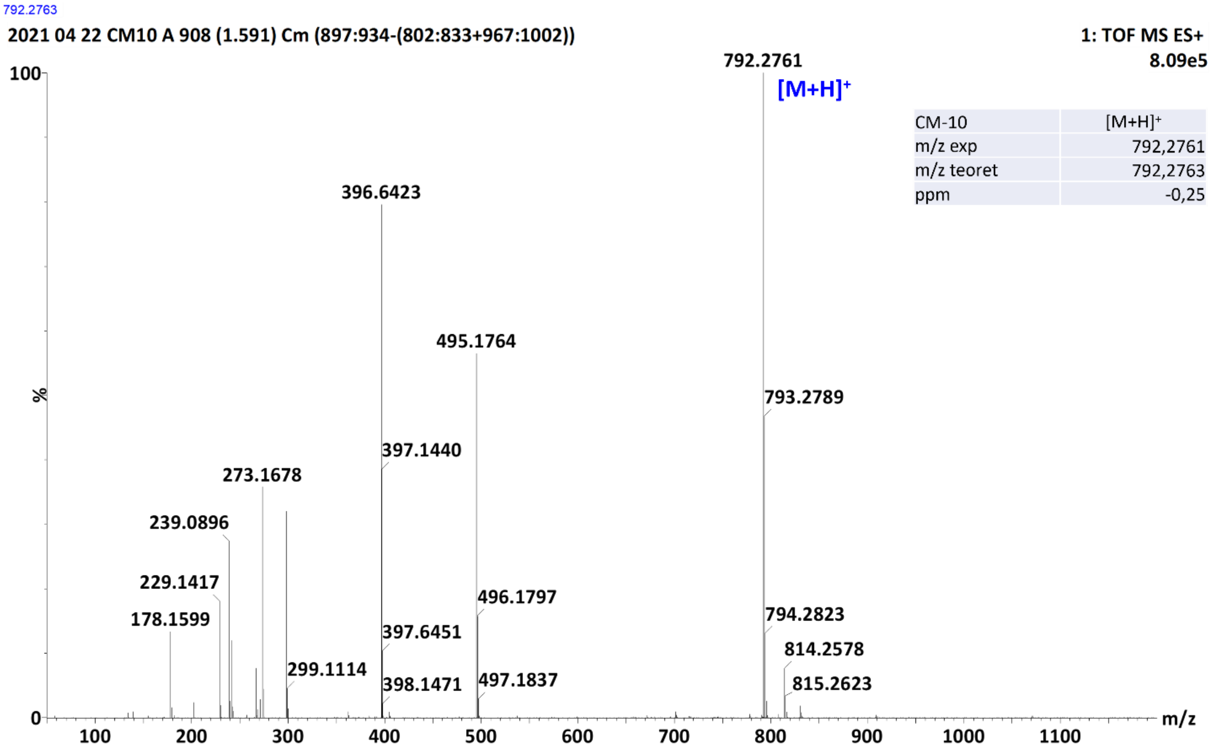

CM-10	[M+H]+
m/z exp	792,2761
m/z teoret	792,2763
ppm	-0,25

Figure S2. ^{1}H NMR spectrum of **3** analyzed in pyridine-d_5 with expanded parts with crucial *J*-couplings highlighted

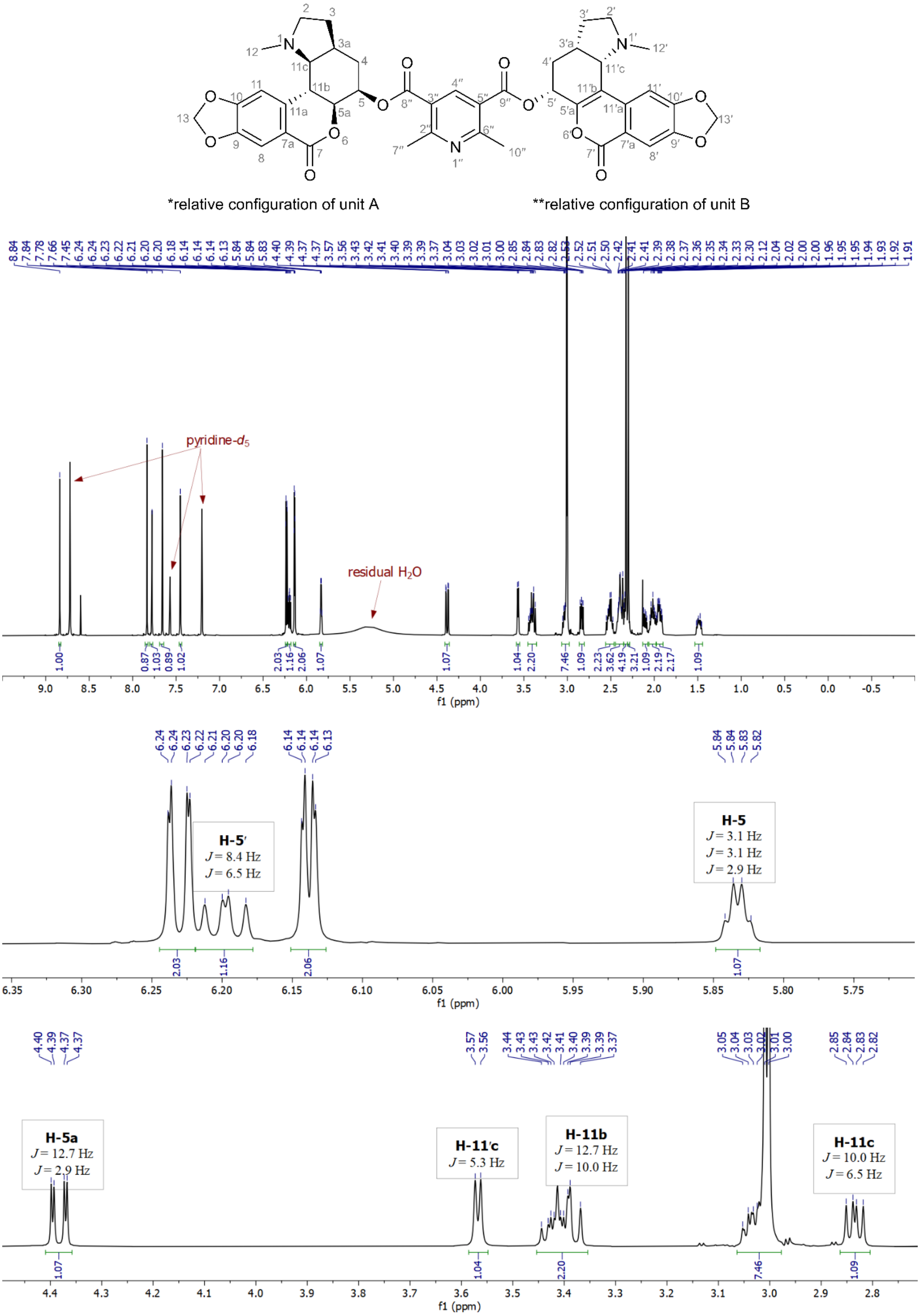

Figure S3. ^{13}C NMR spectrum of **3** analyzed in pyridine-d_5

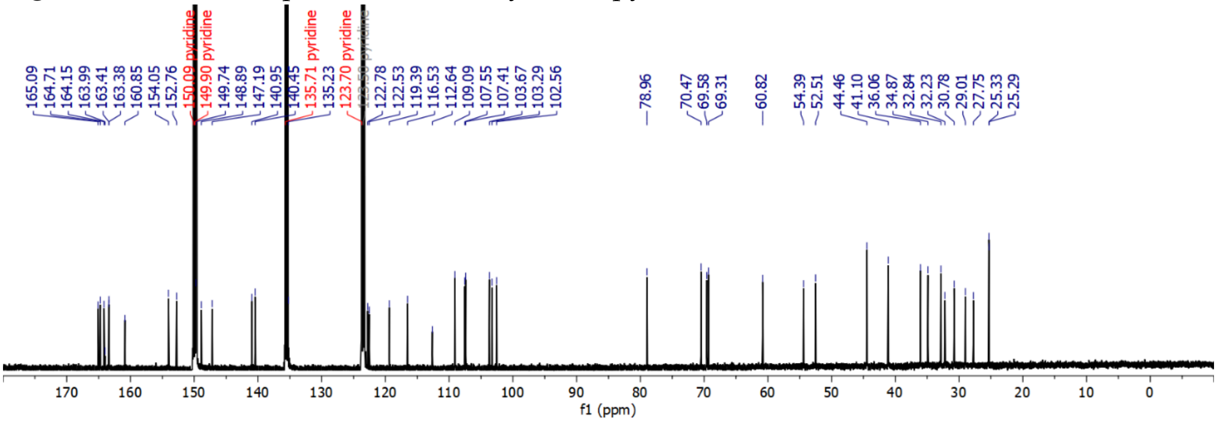

Figure S4. gHSQC spectrum of **3** analyzed in pyridine-d_5

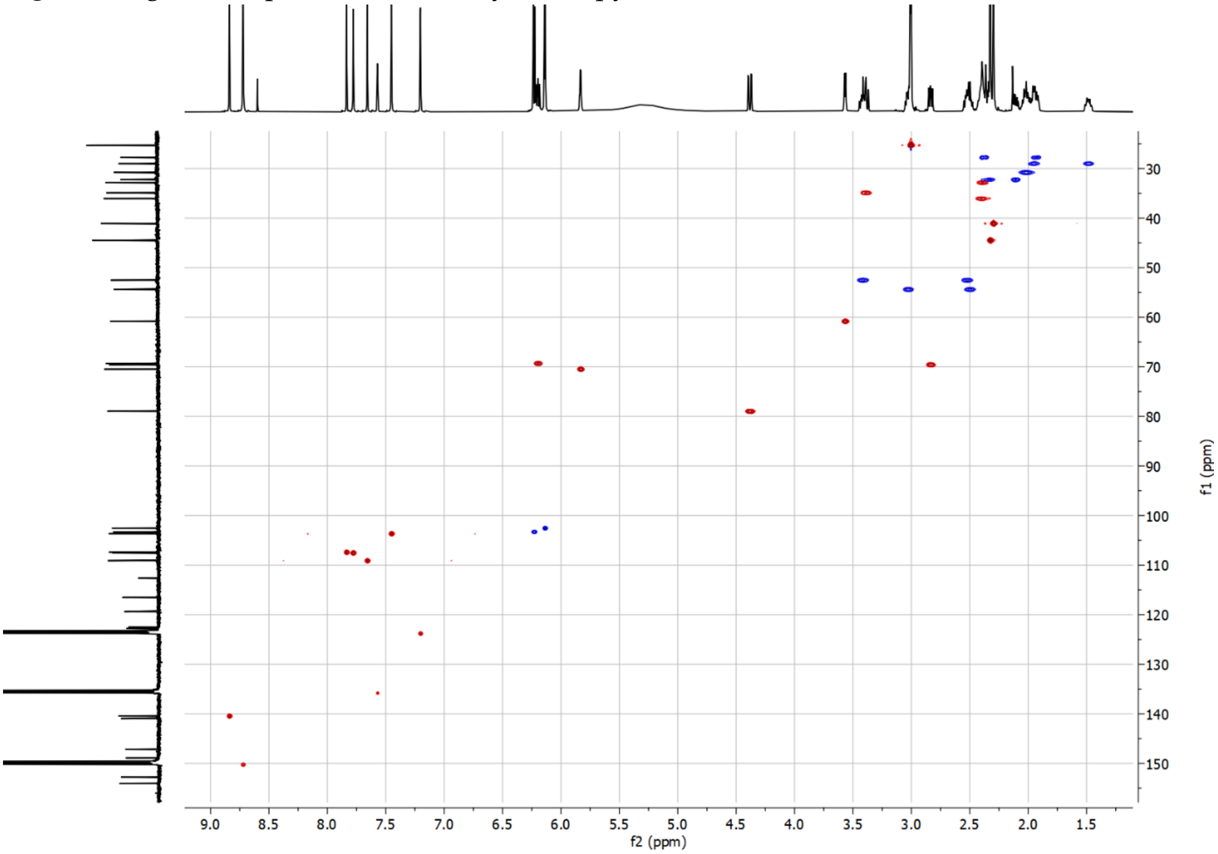

Figure S5. gCOSY spectrum of **3** analyzed in pyridine-*d*5

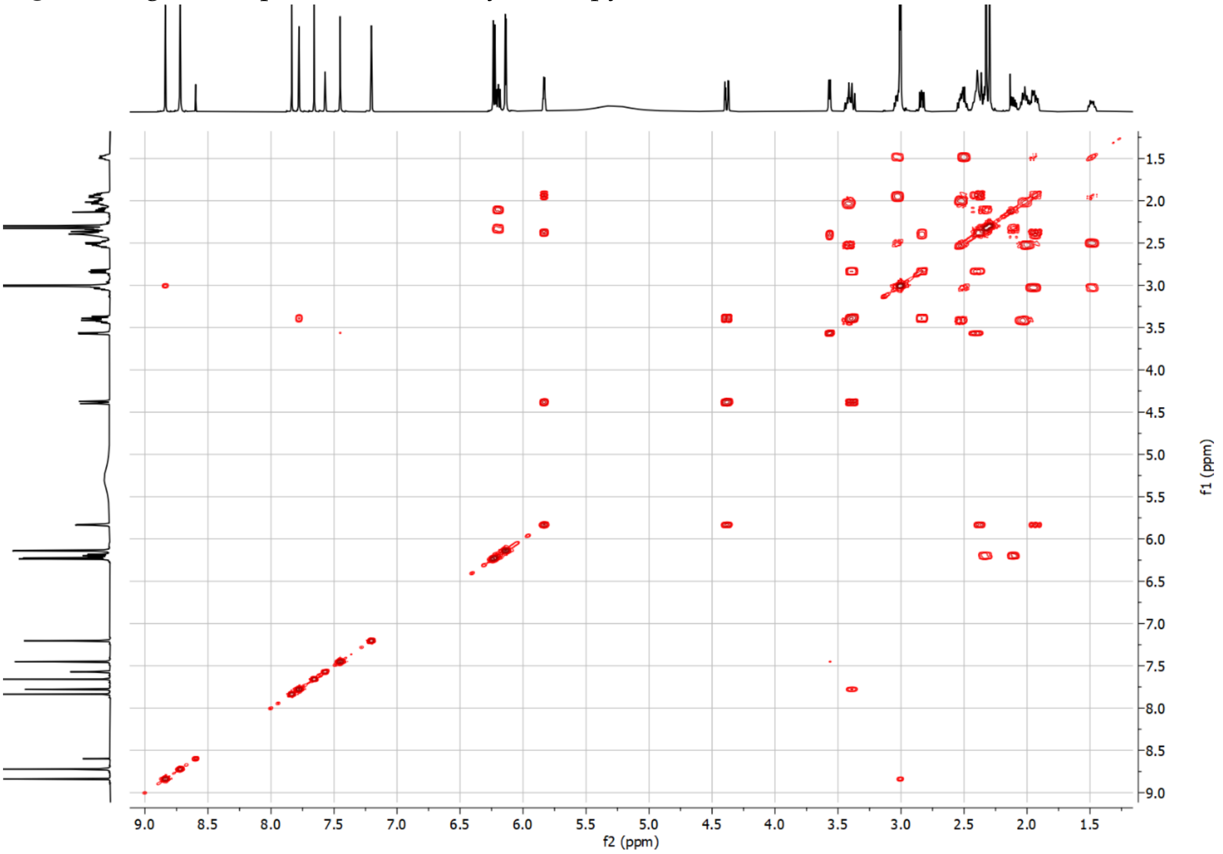

Figure S6. H2BC spectrum of **3** analyzed in pyridine-*d*5

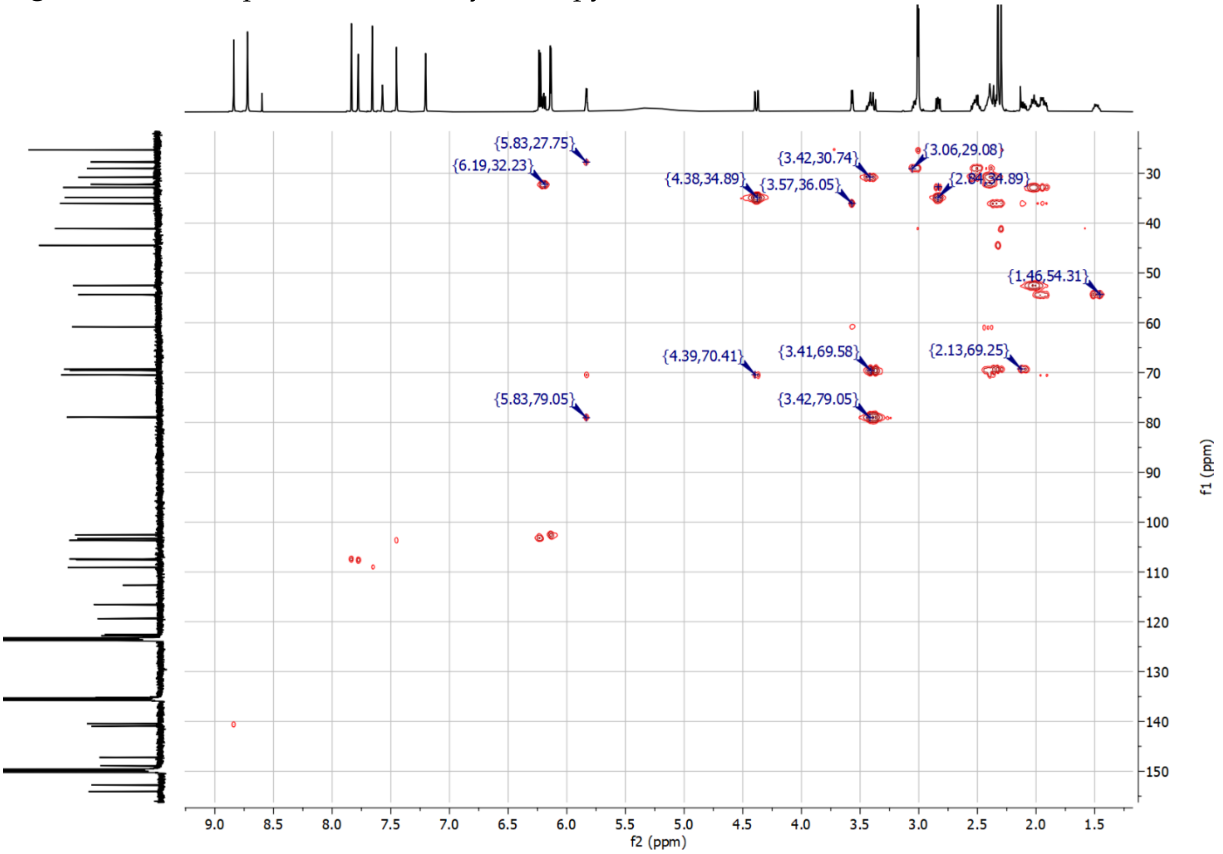

Figure S7. gHMBCAD spectrum of **3** analyzed in pyridine-d_5

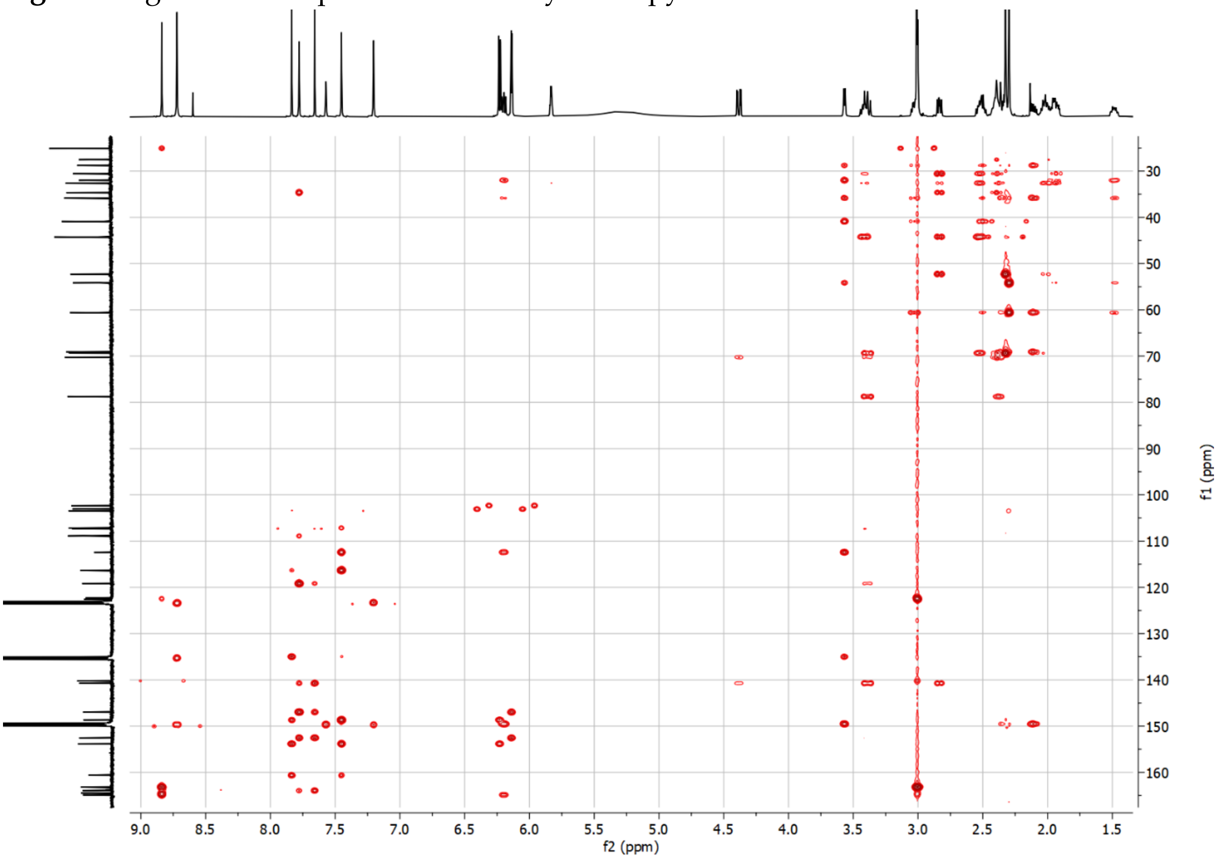

Figure S8. NOESY spectrum of **3** analyzed in pyridine-d_5

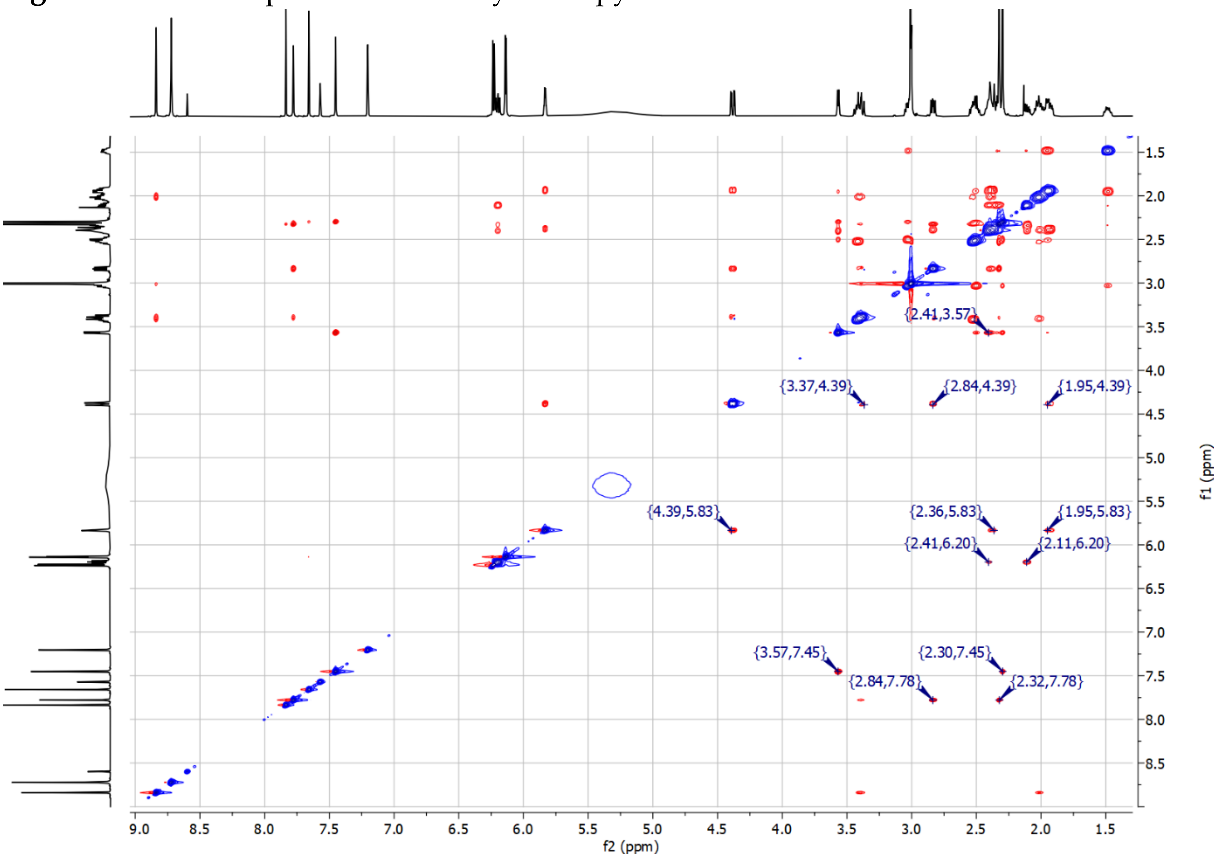

Table S1. Comparison of ^{1}H NMR data of <u>unit A</u> of **3** with the structurally related cliniatine C

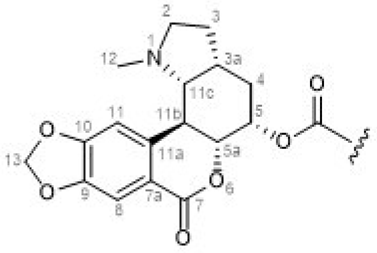

unit A of 3

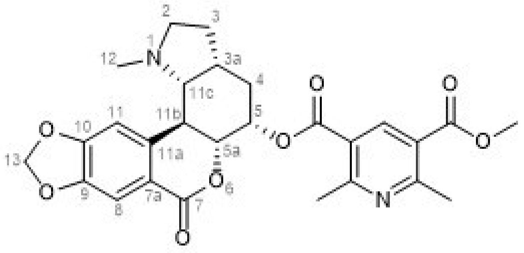

cliniatine C

position	**3**	**cliniatine C** [1]	*type*
	δ_H (500 MHz, C_5D_5N)	δ_H (600 MHz, $CDCl_3$)	
2	3.46–3.38, m<br>2.57–2.46, m	3.37, m<br>2.66, m	CH_2
3	2.08–1.97, m	2.16, m<br>1.94, m	CH_2
3a	2.44–2.35, m	2.66, m	CH
4	2.44–2.35, m<br>1.93–1.89, m	2.48, brd (16.2)<br>2.01, m	CH_2
5	5.83, ddd (3.1, 3.1, 2.9)	5.63, ddd (3.0, 3.0, 2.9)	CH
5a	4.38, dd (12.7, 2.9)	4.29, dd (12.2, 2.9)	CH
7			C=O
7a			C
8	7.66, s	7.50, s	CH
9			C
10			C
11	7.78, s	7.79, s	CH
11a			C
11b	3.39, dd (12.7, 10.0)	3.32, dd (12.2, 9.3)	CH
11c	2.83, dd (10.0, 6.5)	3.04, dd (9.3, 7.2)	CH
12	2.33, s, overlap	2.59, s	CH_3
13	6.14, d, overlap (3.9)<br>6.14, d, overlap (3.9)	6.06, m<br>6.04, m	CH_2

Table S2. Unit B ^{1}H and ^{13}C NMR data compared to C5-epimers from synthesis of narseronine

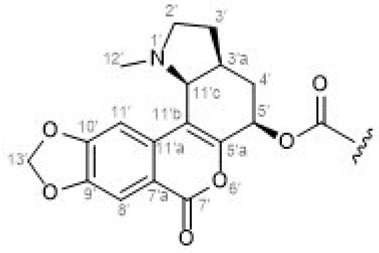

unit B of 3
(3a*R*,5*S*,11c*R*)-isomer

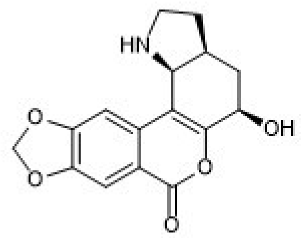

(3a*S*,5*R*,11c*S*)-isomer

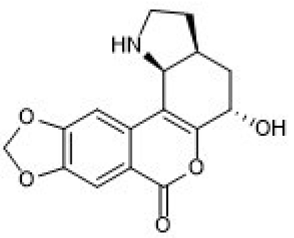

(3a*S*,5*S*,11c*S*)-isomer

^{1}H NMR data δ_H			^{13}C NMR data δ_C		
**3** (C_5D_5N)	5*R*-isomer [2] ($CDCl_3$)	5*S*-isomer [2] ($CDCl_3$)	**3** (C_5D_5N)	5*R*-isomer [2] ($CDCl_3$)	5*S*-isomer [2] ($CDCl_3$)
7.84, s, 1H	7.57, s, 1H	7.33, s, 1H	160.9	161.3	161.3
7.45, s, 1H	7.34, s, 1H	7.17, s, H	154.1	154.1	153.7
6.23, d, overlap (6.5), 1H	6.10, m, 2H	6.06, s, 1H	149.7	152.6	152.3
6.23, d, overlap (6.5), 1H	**4.65, dd (12.0, 6.0), 1H**	6.03, s, 1H	148.9	148.2	148.1
**6.20, dd (8.4, 6.2), 1H**	3.86, d (3.0), 1H	**4.43, s, 1H**	135.2	135.0	134.6
3.57, d (5.3), 1H	3.32, m, 1H	3.76, d (6.0), 1H	116.5	115.3	115.6
3.07–2.98, m, 1H	3.05, m, 1H	3.25, m, 1H	112.6	?*	110.9
2.57–2.46, m, 1H	2.43, m, 1H	3.01, m, 1H	107.4	107.4	107.1
2.44–2.35, m, 1H	2.15–2.02, m, 3H	2.69, m, 1H	103.7	103.5	103.3
2.35–2.28, m, 1H	1.78–1.63, m, 2H	2.13, m, 1H	103.3	102.3	102.3
2.30, s, overlap, 3H		1.90–1.71, m, 2H	69.3	65.9	64.3
2.14–2.08, m, 1H		1.59, m, 1H	60.8	57.1	56.4
1.97–1.93, m, 1H			54.4	46.0	46.1
1.53–1.44, m, 1H			41.1	**35.7**	**32.7**
			**36.1**	**33.1**	**32.5**
			**32.2**	31.8	31.2
			29.0	-	-

* "one signal obscured or overlapping"; but the Supplementary Material[2] shows a signal around 110 ppm that is just not marked.

Important signals in bold. Although the substitution between the compared 5-epimers and **3** is different, as is the solvent used, which affects the chemical shift, the spin-spin interactions can be compared. In ^{1}H NMR data interpretation, the large *J*-constant corresponds to *cis*-orientation methine hydrogens of this scaffold. On the other hand, broad singlet represents *trans*-orientation of H-5/H3a. Interestingly, with ^{13}C NMR chemical shift, the influence of 5-epimerization is therefore more significant at the C-3a and C-4 positions than at C-5.

Biological assay in detail

***h*AChE and *h*BuChE inhibition assay**

The inhibitory activities of prepared compounds and standards against human recombinant AChE (E.C. 3.1.1.7) and human plasma BuChE (E.C. 3.1.1.8) were determined using modified Ellman's method (Ellman et al., 1961) and expressed as IC_{50} (the concentration of the compound that is required to reduce 50% of cholinesterase activity). Human recombinant AChE, phosphate buffer (PB, pH = 7.4), 5,5′-dithio-bis(2-nitrobenzoic) acid (Ellman's reagent, DTNB), acetylthiocholine (ATCh), butyrylthiocholine (BTCh), and other used compounds were purchased from Sigma-Aldrich (Prague, the Czech Republic). Human plasma was used as a source of BuChE and was prepared from heparinized human blood. Blood was centrifuged for 20 minutes (4 °C, 2300 × g) by Hettich Universal 320R centrifuge. The plasma was separated and stored at −80 °C. During the measurement, 96-well microplates from polystyrene (ThermoFisher Scientific, Waltham, MA, USA) were used.

The solutions of the corresponding cholinesterase in PB were prepared up to the final activity 0.002 U/μL. The assay medium (100 μL) consisted of cholinesterase (10 μL), DTNB (20 μL of 0.01 M solution), and PB (40 μL of 0.1 M solution). The solutions of the tested compounds (10 μL of different concentrations) were pre-incubated for 5 minutes in the assay medium and then a solution of the substrate (20 μL of 0.01 M ATCh or BTCh iodide solution) was added to initiate the reaction. The increase of absorbance was measured at 412 nm using Multimode microplate reader Synergy 2 (BioTek Inc., Winooski, VT, USA). For the calculation of the resulting measured activity (the percentage of inhibition I) following formula was used:

$$I = \left(1 - \frac{\Delta A_i}{\Delta A_0}\right) \times 100$$

where ΔA_i indicates absorbance change provided by adequate enzyme exposed to corresponding inhibitor and ΔA_0 indicates absorbance change when a solution of PB was added instead of a solution of inhibitor. Software Microsoft Excel (Redmont, WA, USA) and GraphPad Prism version 6.07 for Windows (GraphPad Software, San Diego, CA, USA) were used for the statistical data evaluation.

References

1. Hirasawa, Y., et al., *Cliniatines A-C, new Amaryllidaceae alkaloids from Clivia miniata, inhibiting Acetylcholinesterase.* J Nat Med, 2022. **76**, p. 171–177. DOI: 10.1007/s11418-021-01570-6

2. Yang, S.X., et al., *A Chemoenzymatic Route to the (+)-Form of the Amaryllidaceae Alkaloid Narseronine.* Australian Journal of Chemistry, 2015. **68**(2): p. 241–247. DOI: 10.1071/CH14520
